# Beneficial Betrayal Aversion

**DOI:** 10.1371/journal.pone.0017725

**Published:** 2011-03-14

**Authors:** Jason A. Aimone, Daniel Houser

**Affiliations:** Interdisciplinary Center for Economic Science, George Mason University, Arlington, Virginia, United States of America; Royal Holloway, University of London, United Kingdom

## Abstract

Many studies demonstrate the social benefits of cooperation. Likewise, recent studies convincingly demonstrate that betrayal aversion hinders trust and discourages cooperation. In this respect, betrayal aversion is unlike socially “beneficial” preferences including altruism, fairness and inequity aversion, all of which encourage cooperation and exchange. To our knowledge, other than the suggestion that it acts as a barrier to rash trust decisions, the benefits of betrayal aversion remain largely unexplored. Here we use laboratory experiments with human participants to show that groups including betrayal-averse agents achieve higher levels of reciprocity and more profitable social exchange than groups lacking betrayal aversion. These results are the first rigorous evidence on the benefits of betrayal aversion, and may help future research investigating cultural differences in betrayal aversion as well as future research on the evolutionary roots of betrayal aversion. Further, our results extend the understanding of how intentions affect social interactions and exchange and provide an effective platform for further research on betrayal aversion and its effects on human behavior.

## Introduction

Many studies[1]–[3] demonstrate the social benefits of cooperation. Likewise, recent studies convincingly demonstrate that betrayal aversion hinders trust and discourages cooperation[4]–[8]. In this respect, betrayal aversion is unlike socially “beneficial” preferences including altruism[9], fairness[10], [11] and inequity aversion[11], [12], all of which encourage cooperation and exchange. To our knowledge, other than the suggestion that it acts as a barrier to rash trust decisions[13], the benefits of betrayal aversion remain largely unexplored. Here we use laboratory experiments with human participants to show that groups including betrayal-averse agents achieve higher levels of reciprocity and more profitable social exchange than groups lacking betrayal aversion. These results are the first rigorous evidence on the benefits of betrayal aversion, and may help future research investigating cultural differences in betrayal aversion[4], [5] as well as future research on the evolutionary roots of betrayal aversion. Further, our results extend the understanding of how intentions affect social interactions and exchange[10] and provide an effective platform for further research on betrayal aversion and its effects on human behavior.

A trusting agent (henceforth an “investor”) making a decision to trust a counterpart (henceforth a “trustee”) faces uncertainty beyond that of a monetary risk of a high or a low payment[13]–[19]. Indeed, trust exposes the investor to the potential emotional cost of learning that their counterpart betrayed their trust. Aversion to the latter is known as betrayal aversion[4]–[9]. Past studies[4]–[8] show that, with all else equal, betrayal aversion reduces willingness to trust. Nevertheless, the research to date has focused only on the negative aspects of betrayal aversion (i.e., that it appears to be a factor that hinders beneficial social exchange). This study provides the first evidence of the adaptive benefits of betrayal aversion.

We use a laboratory trust game experiment[20], [21] with human subjects to test the hypothesis that reciprocation is more frequent when investors exhibit betrayal aversion. Note that if this is the case, as our evidence supports, then investors who are known to be betrayal-averse face relatively more profitable social exchange opportunities. Consequently, investors lacking betrayal aversion have an incentive to adopt behavior consistent with that of betrayal-averse investors, through learning or imitation, to reap the monetary benefits.

Trustees known to be sympathetic towards betrayal-averse investors have their own advantage over non-sympathetic trustees through the social selection process[2]. They are more attractive exchange partners due to higher rates of reciprocation (see Supporting Text S1 for model details.) As such, the equilibrium rates of reciprocation within betrayal-averse groups are expected to increase over time. This leads to the expected formation of higher social norm rates of reciprocation, supporting and bolstering the evolution and success of cooperation. It follows that groups with betrayal-averse investors are expected to hold an evolutionary advantage over otherwise equivalent groups lacking this betrayal-aversion.

**Figure 1:**
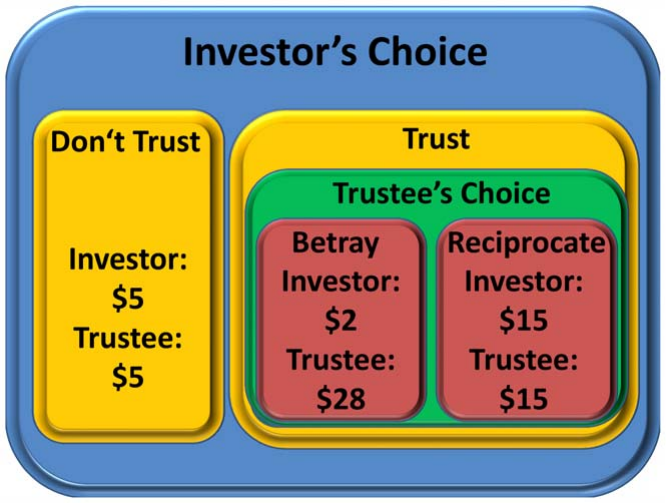
The Binary Trust Game.

Our investigation of the benefits of betrayal aversion begins with saliently rewarded laboratory trust games[8], as in the binary trust game illustrated in Figure 1. In these trust games, we randomly assign subjects to the role of either investor or trustee and then randomly assign a specific counterpart to the opposite role. The investor decides whether to split a $10 endowment evenly with their trustee counterpart ($5 each) or to “trust” the trustee. If the investor chooses to trust, the $10 triples to $30, and the trustee chooses from two options: (i) “reciprocate”: split the $30 evenly with the investor ($15 each); or (ii) “betray”: split the $30 unevenly with the investor ($28 to the trustee and $2 to the investor.) This game is “one-shot,” meaning that subjects play the game only once, with only one counterpart. In addition to this baseline trust game, the KNOW treatment, we review two additional trust game treatments, the DONTKNOW1 and OPTION treatments (which appeared previously in Aimone and Houser 2009[8]) that form the baseline for the current study. We then present a new treatment, DONTKNOW2, which allows us to study the effect of betrayal aversion on trustees' decisions.

**Figure 2:**
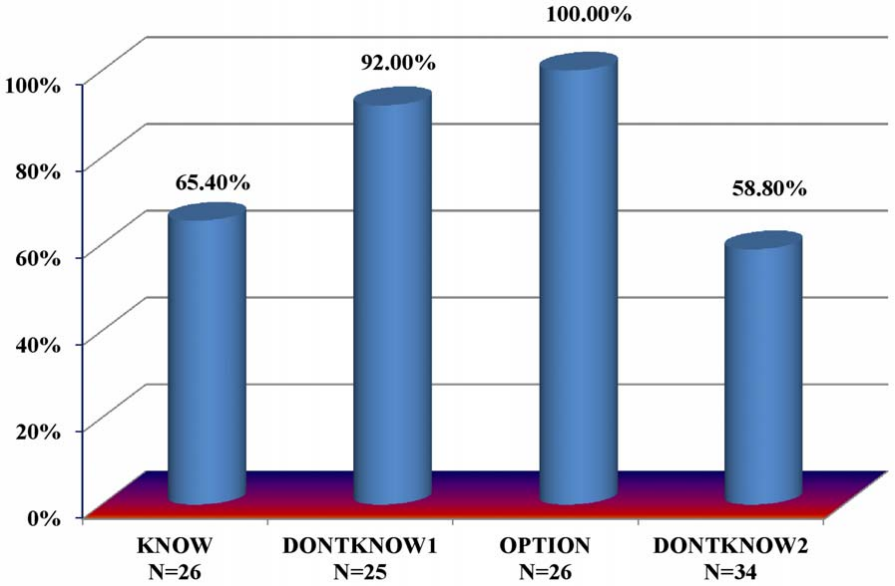
Trust by Treatment. Bars indicate the percentage of investors choosing the trust gamble.

In the KNOW treatment, 65.4% of investors choose to trust their trustee counterpart. We next used a modified trust game, DONTKNOW1, which removes the risk of emotional disutility associated with betrayal. In particular, if the investor chooses to trust, we pay the investor based upon a separate random draw, without replacement, from the pool of trustee decisions, instead of payment based upon the decision of their specific counterpart. Their payment still reveals that either some trustee chose to reciprocate (if they are paid $15), or that some trustee chose to betray trust (if they are paid $2). The key manipulation is that the payment amount no longer reveals whether the investor's own counterpart chose to betray trust. Figure 2 shows that significantly more investors trust in DONTKNOW1 (92.0%, Mann-Whitney p = 0.022) when the emotional risk has been removed.

We find maximum trust, 100%, to occur in the OPTION treatment, where investors can choose whether or not to be exposed to the knowledge of betrayal, and investors have the option to be paid based upon either their specific counterpart's decisions or the random draw from the pool of trustee decisions.

The results of DONTKNOW1 and OPTION show that investors are generally willing to assume the monetary risks of a trust gamble, but many are unwilling to assume the emotional risks associated with learning their trusted counterpart chose to betray. Since these treatments are identical from the trustee's perspective, we pool trustees' decisions, observing that 66.3% of trustees betray trust. At this rate of betrayal, the expected value of trusting, $6.38, is greater than the safe option, $5.00, making trust a monetarily profitable venture. When an investor chooses not to trust, the monetary welfare gains from social exchange are not realized. As such, the results appear to suggest that societies without betrayal aversion would have an evolutionary advantage, due to their relatively greater willingness to engage in profitable social exchange when exposed to an emotional risk of betrayal. This is, of course, inconsistent with the presence of betrayal aversion. To shed light on the adaptive benefits of betrayal aversion, we conduct a new treatment, the DONTKNOW2 treatment, which examines the effect of betrayal aversion on trustee behavior.

## Results

Our new treatment, DONTKNOW2, is identical to DONTKNOW1, aside from the fact that both investors and trustees know that the exchange environment shields investors from the knowledge of betrayal, i.e. the institution is common knowledge. In DONTKNOW2, a trustee's decision to reciprocate or betray still has the same expected monetary impact upon each of the anonymous random counterparts, but a decision to betray no longer exposes their assigned investor counterpart to the negative emotions associated with betrayal. Therefore, to an other-regarding trustee, an act of betrayal is now relatively more rewarding. The relatively increased rewards could come through several channels, depending upon the form of other-regarding preferences of each trustee, but are always tied to the reduction of emotional disutility associated with betrayal knowledge. For example, an altruistic trustee, or a guilt-averse trustee[22], would be more willing to betray since they would know their “bad” action, betrayal, has only expected monetary effects without disutility associated with an added negative emotional reaction. Similarly, a change in “moral wiggle-room” [23] could increase trustees' willingness to betray only if there was an expected difference in emotional losses to investors.

**Figure 3:**
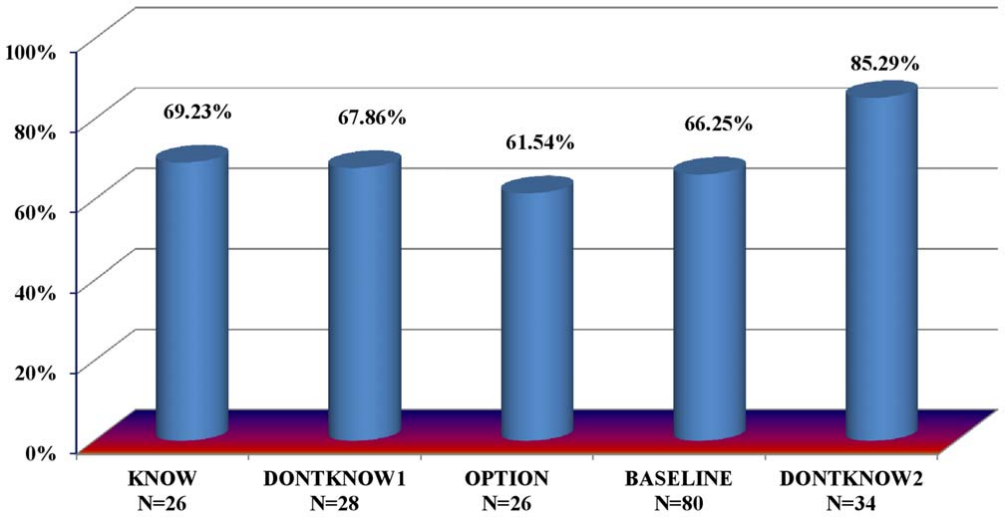
Betrayal by Treatment. Bars indicate the percentage of trustees choosing to betray trust.

A comparison of betrayal in DONTKNOW2 to betrayal in KNOW, DONTKNOW1, and OPTION (the BASELINE treatments in Fig. 3) provides a test of whether trustees are averse to betraying a specific investor counterpart. The 85.3% betraying in DONTKNOW2 is significantly greater than the 66.3% (MW, p = 0.039) we observed in BASELINE. The results indicate that trustees are, as hypothesized, more willing to betray when the investor's knowledge of betrayal has been removed from the social exchange environment.

While investors are equally shielded from the knowledge of personal betrayal in both DONTKNOW1 and DONTKNOW2, neither treatment shields investors from the monetary risk associated with the trust gamble. If investors in DONTKNOW2 expect lower rates of reciprocation than investors in DONTKNOW1, then we would expect monetary risk aversion to lead to lower rates of trust from investors in DONTKNOW2 than from investors in DONTKNOW1. We find that this is indeed the case.

Note that the additional 19% chance of betrayal in DONTKNOW2 drops an investor's expected return from trusting to $3.91, an expected loss compared to the $5 sure payout of not trusting. Accordingly, significantly fewer investors trust in DONTKNOW2, (58.8%) compared to DONTKNOW1 (92.0%; MW, p = 0.005), where investors were also not susceptible to their betrayal aversion and the expected return of trusting was $6.38. This difference in rates of trust illustrates that while betrayal aversion is no longer affecting decision-making, monetary risk aversion is still affecting decision-making in DONTKNOW1 and DONTKNOW2.

## Discussion

A willingness to cooperate in mutually beneficial social exchange is a great advantage for groups holding other-regarding preferences, especially when social exchange requires trust or lacks beneficial reputation and repeat game effects, as in our one-shot experiments. Our experiments suggest that betrayal aversion has duel effects. First, betrayal aversion has a “negative” effect of reducing investor willingness to trust given an expected rate of reciprocation. Second, the presence of betrayal-averse agents has a beneficial effect of interacting with the other-regarding preferences of trustees to increase rates of reciprocation and cooperation. As a result, the expected profitability of social exchange requiring trust is greater in the presence of betrayal-averse agents than without. The increased expected monetary return in these environments leads to an increased willingness to engage in social exchange requiring trust. This, in turn, offsets the decreased willingness to trust caused by betrayal aversion.

Our results draw attention to the importance of understanding all sides of a social interaction when investigating intentions, not just the effects of intentions on response behavior typically identified by using randomization devices such as computers and dice[10], [16], [24]. Trustees, like proposers in ultimatum games who reduce offers when the likelihood of rejection decreases, consider not only their own position, but the position of their counterpart.[25] In our experiment, trustees' increased willingness to reciprocate trust in the presence of betrayal-averse agents indicates that trustees generally anticipate and respond to the expected negative emotional responses investors have to intentional acts of betrayal. It is unlikely that betrayal aversion is unique in this regard. These expectations are particularly important for comparative institution studies. Any institutions designed to reduce the negative effects of betrayal aversion must also be designed to maintain the beneficial heightened levels of reciprocation also associated with betrayal aversion[Aimone and Houser, 2010].

This study is a first step in understanding the beneficial side of betrayal aversion. Future neurological studies can provide evidence for whether betrayal aversion has genetic foundations, as suggested by recent research on oxytocin[13], or whether cultural differences lead to the betrayal aversion differences observed between countries[4], [5]. Additionally, future research could provide evidence to distinguish between aversion to being the victim of a personal betrayal and aversion to the knowledge that one's trust was betrayed, a distinction absent from past studies of betrayal aversion.

## Materials and Methods

This project was approved by the Human Subjects Review Board of George Mason University. All 228 subjects signed informed consent prior to participating. Our goal is to create an experiment that replicates the design of Aimone and Houser (2009)[8], henceforth “AH,” as precisely as possible, but run the DONTKNOW treatment in a common knowledge environment. We explain the AH methods first, and then our extension treatment. A transcript of the instructions for each treatment can be found in Supporting Text S2 and S3.

### The Trust Decision

The trust game illustrated in Figure 1 (the “KNOW” treatment in AH) acts as a foundation for the treatments in the experiment. A human investor (Player 1) and a human trustee (Player 2) make decisions simultaneously. Each investor decides whether to “trust,” i.e, allow the trustee to choose a division of $30, or not to “trust,” i.e., divide $10 evenly with their trustee counterpart ($5 each.) If the investor chooses to “trust” then payments are determined by the trustee's decision between two options, betrayal or reciprocation. If the trustee chooses to betray the investor's trust, then the investor is paid $2 and the trustee $28. However, if the trustee chooses to reciprocate trust, then the $30 is divided evenly ($15 each.) Note that the game is one-shot, the instructions use neutral language, and the game tree is not distributed to subjects.

### OPTION-TO-KNOW and DONTKNOW1 Treatments

The “OPTION-TO-KNOW” (OPTION) treatment is similar to KNOW. The instructions inform investors that they have both a randomly-assigned human counterpart, as well as a randomly-assigned computer-generated “decision” in the experiment. In addition to the trust and don't trust options, each investor also has a third option to choose to be paid according to the randomly assigned computer's decision. In the event the investor chooses the third option (the computer option), the investor's counterpart trustee is paid based upon his/her own decision, just as the trustee would have been paid in the event the investor chose the trust option. Since the computer decision is a draw (without replacement) from a pool identical to that specific session's pool of trustees' decisions, the expected monetary gain or loss to the investor is also the same whether they choose to payment based on the computer's decision or they choose to trust their counterpart trustee. The difference is that they do not learn whether their trustee counterpart chose to betray or reciprocate. All investors complete a quiz over the instructions and procedure before making their decision (in Supporting Text S2.)

The DONTKNOW1 treatment is identical to the OPTION treatment with the exception that the investor has only the first and third options, i.e.., don't trust or be paid based upon the computer's decision. See Supporting Text S2 for the details on how the computer's random draw without replacement occurred. These details are in the instructions as well.

### Common Knowledge and Non-Common Knowledge Procedures

In AH, subjects in the role of investor sat in “room A” and subjects in the role of trustees sat in a different “room B.” Each room had an equal number of subjects (say N, which was either eight or ten) who were seated at visually (but not acoustically) isolated desks. Subjects received either a “room A” set of instructions or a “room B” set of instructions respectively. A monitor in each room read the instructions aloud to the subjects. All subjects completed a short graded quiz to ensure they understood their room's instructions and a monitor read aloud the questions and answers after all subjects successfully completed the quiz. Each investor then drew a number (from a box containing the numbers one through N.) This number, drawn without replacement, would match them with a specific trustee counterpart in “room B” who was previously randomly assigned that number based on where they sat in the lab. In the DONTKNOW1 and OPTION treatments, this number also randomly matched the investor with one of the ten computer decisions also previously randomly assigned a number between 1 and N. After making decisions (as described in the treatment sections above), subjects filled out a general questionnaire and were paid in cash privately, immediately before leaving the laboratory. Note that investors and trustees sat divided into two rooms, so instructions were not common knowledge.

We implemented the common knowledge version of DONTKNOW1 (DONTKNOW2) in the same manner as above by making only a few changes. First, monitors directed “room A” and “room B” subjects into the same room, instead of two different rooms. Second, as each subject entered the room, they drew (out of a common stack) a copy of both the investors' (“room A”) instructions and trustees' (“room B”) instructions. One monitor read aloud the investors' instructions and a second monitor read aloud the trustees' instructions to all subjects (order alternated between sessions.) Immediately following each set of instructions, all subjects completed a graded quiz for that set of instructions. After finishing the graded quiz, a monitor read the questions and answers for the quiz aloud to all subjects. With these procedures, both the trustees' instructions and the investors' instructions were common knowledge to all subjects. By making both trustees and investors take a graded quiz on both sets of instructions (with answers read aloud to all subjects,) it was common knowledge that all subjects had a basic understanding of all decisions, instructions, and procedures at work in the experiment.

## Supporting Information

Text S1 Model of Betrayal Aversion.
(DOC)

Text S2 Instructions: Aimone and Houser 2009 Treatments.
(DOC)

Text S3 Instructions: DONTKNOW2 **(**Common Knowledge) Treatment.
(DOC)
