# Supplementary material for: Beneficial Betrayal Aversion
Source: PLoS One. 2011 Mar 14;6(3):e17725. doi: 10.1371/journal.pone.0017725 (PMC3056706; doi:10.1371/journal.pone.0017725)
Supplement: Text S1 — Model of Betrayal Aversion. (DOC) [file pone.0017725.s001.doc]

**Supporting Text S1**

**Model of Betrayal Aversion**

Experimental research on fairness, equality, altruism, and similar topics[11,22] suggest that the utility of a trustee in a trust game[14] is dependent on the beliefs and outcomes of his/her counterpart investor. Betrayal of trust decreases the monetary payoff (and utility) of an investor, and thus an other-regarding trustee (with a decreasing own-marginal utility of wealth) may be willing to reciprocate a positive amount to increase the investor’s utility. If investors are betrayal averse (as AH demonstrate,) then betrayal triggers negative utility separate from the lower utility from decreased monetary earnings. An other-regarding trustee would then also experience reduced utility through how the investor’s utility enters the trustee’s utility function.

Consider a version of the standard binary investment game as in AH. Let π_i_ be the final payoffs for i = {y, x}, where y is an investor and ‘x’ is ‘y’s randomly matched counterpart trustee, both drawn randomly from the same pool. In the AH trust game, ‘y’ decides whether to take the safe-option, divide $10 equally and set payoffs (π_y_,π_x_)= ($5,$5), or to trust, which triples total payouts and allows ‘x’ to divide $30. If ‘y’ trusts, then ‘x’ has to decide between betraying trust, β_x_ = 1,which sets (π_y_,π_x_)= ($2,$28), and reciprocating trust, β_x_ = 0, which would set (π_y_,π_x_)= ($15,$15). As standard, calling this investment game a “trust” game, requires the assumption that ‘y’ prefers the ($15,$15) outcome to the ($5,$5) outcome, which are both preferred to the ($2,$28) outcome. ‘Y’ must also expect ‘x’ to understand this is the case.

Since choosing to betray results in a large monetary payoff to ‘x’, if ‘y’ trusts, a non-other-regarding trustee will always choose β_x_ = 1 in a one-shot trust game, and the investor will subsequently not trust. For other-regarding trustees,’ though, we can express the utility function of ‘x’ as U_x_(π_x_, φ*U_y_): a function of final payoff to ‘x’ (π_x_), the utility of ‘y’ (U_y_), and an altruism parameter (φ є [0,1).) φ = 0 would imply a completely non-altruistic person. We use altruism here as a specific form of other-regarding preferences. Other forms of other-regarding preferences could also be substituted in place of altruism.

A payoff of π_y_ = $2 informs ‘y’ that ‘x’ chose to betray trust (β_x_ =1) while a payoff of π_y_ = $15 informs ‘y’ that ‘x’ chose to reciprocate trust (β_x_ = 0). If ‘y’ chooses the safe option, then y has no knowledge of ‘x’s decision. The definition of betrayal aversion implies that information of ‘x’s decision to betray (β_x_ = 1) negatively affects ‘y’s utility (U_y_), if ‘y’ has decided to trust. The utility of investor ‘y’ can be written as:

Eq. 1 U_y_(π_y_=$2, β_x_ = 1| betrayal averse) < U_y_(π_y_=$2, β_x_ =1| not betrayal averse)

Since betrayal aversion (henceforth ‘B.A.’) does not come into play if ‘x’ chooses to reciprocate (β_x_ = 0):

Eq. 2 U_y_(π_y_=$15, β_x_ = 0 | B.A.) = U_y_(π_y_=$15, β_x_ = 0| Not-B.A.)

Let γ be the probability that ‘x’ thinks ‘y’ is betrayal averse. ‘X’s utility is then:

Eq. 3 U_x_(π_x_, φ*[γ*U_y_(π_y_, β_x_ |B.A.) + (1-γ)*U_y_(π_y_, β_x_ |Not-B.A.)])

A trustee’s decision to reciprocate or betray is then based on the sign of:

Eq. 4 [U_x_(π_x_=$28, φ*[γ*U_y_(π_y_=$2, β_x_ = 1|B.A.) + (1-γ)*U_y_(π_y_=$2, β_x_ = 1|Not-B.A.)])] </>/= [U_x_(π_x_=$15, φ*(U_y_(π_y_=$15, β_x_ = 0)]

Equation 4 shows how betrayal and reciprocation change as altruism and expectations about others change. First, as altruism increases, φ increases and thus the right side of Eq. 4 increases relative to the left side. Second, if the expected probability that a person is betrayal averse increases, then the average expected utility of betraying trust decreases. This is seen in how an increase in γ in eq. 8 reduces the value of left side of the equation, but does not affect the right side. Additionally, if the expected disutility associated with betrayal aversion were to increase, the left side of Eq. 4 would again decrease, without decreasing the right hand side. This model suggests that the frequency of betrayal will decrease as the likelihood of interacting with a betrayal averse person increases and as the disutility associated with betrayal aversion increases.

A decreased incidence of betrayal implies an increased expected monetary return from social exchange, which in turn should support participation in social exchange (trust) by investors. The presence of betrayal-averse agents then becomes a phenomenon that potentially promotes reciprocation and encourages social exchange. These benefits of betrayal aversion help explain the evolutionary presence of betrayal aversion (a population of betrayal-averse agents can grow more efficiently than a population of non-betrayal-averse agents due to beneficial social exchange.) This type of argument could also explain why we see rejection of unfair offers in private impunity games [26].

Institutions designed to eliminate the knowledge of betrayal, like AH, might initially appear to be helpful to betrayal-averse investors and increase trust. As shown in the model above, though, this institution would be expected to lead altruistic trustees to betray more often, since investors could no longer feel the emotional pangs of betrayal. The increases in trust from the removal of betrayal knowledge are then offset by the increase in expected probability of trustees choosing the betrayal option. Our laboratory experiment tests whether betrayal increases when the exchange institution removes betrayal aversion from the trust environment. The experiment also tests whether the decreases in trust, if any, associated with the expected increase in monetary risk, are sufficient to offset the increases in trust associated with the removal of betrayal aversion from the environment.
