# Supplementary material for: Beneficial Betrayal Aversion
Source: PLoS One. 2011 Mar 14;6(3):e17725. doi: 10.1371/journal.pone.0017725 (PMC3056706; doi:10.1371/journal.pone.0017725)
Supplement: Text S2 — Instructions: Aimone and Houser 2009 Treatments. (DOC) [file pone.0017725.s002.doc]

**Supporting Text S2**

**Room A (Investors) Instructions: OPTION-TO-KNOW Treatment[[1]](#footnote-2)**

Thank you for participating in today’s experiment. You’ve earned a $7 show-up bonus for participating. In reading and following the instructions below, you have the potential to earn significantly more. You have been randomly assigned to **Room A.** You will also be randomly and anonymously assigned to a person in **Room B.** Your counterpart will not be told your name, and you will not be told his/her name.

**How you are matched with your counterpart:**

Each of the 10 Room A persons will be matched with a different Room B counterpart for the entire experiment. The experimenter will bring around a box with the numbers 1 through 10 inside. The number you draw will assign you to one of the 10 counterparts in Room B (B1 through B10 coinciding with the numbers 1 through 10 in the box). The number also matches you with one of the 10 computer number decisions (coinciding with numbers 1 through 10 in the box).

**Your Decision:**

You have three options for how the earnings for you and your counterpart will be determined in today’s experiment. You must choose exactly one of the following three options:

- You receive $5 and your counterpart receives $5.
- Both you and your counterpart are paid based on his/her decision between **“U”** ($15 for you and $15 for him/her) and **“D”** ($2 for you and $28 for him/her).
- Your counterpart is paid according to his/her decision between **“U”** and **“D”**, and you are paid based on a computer’s choice between either **“U”** or **“D”**.

You will not be told what the computer’s decision was, or what your counterpart’s decision was, unless you choose that earnings option.

**Room B Decision:** (The instructions given to your counterpart)

You will be anonymously assigned to a Room A counterpart who drew your number randomly from a box with the numbers 1 through 10 inside. This person will be your counterpart for the entire experiment. Your counterpart will make a decision that can affect your earnings in today’s experiment. He or she can choose for both of you to be paid $5. Another possibility is that he/she will let you determine both of your payoffs. If he/she chooses this option and you choose **“U”**, then you get paid $15 and he/she gets paid $15. If you choose **“D”**, then you get paid $28 and he/she gets paid $2. Your payoff will be determined in one of these two ways. Your counterpart can choose only one of the earnings methods. We will ask you to make your decision on **“U”** or **“D”** at the same time that your counterpart makes his or her choice. Your decision will only determine your payoff if your counterpart did not choose the option to give you $5.

**Computer’s Decision:**
After the Room B participants make their decisions, the computer will assign either **"U"** or **"D"** to each of the ten numbers. The computer has been programmed to assign dollar values to each of the 10 numbers in the box according to the decisions made by the Room B participants. What this means is that the number of **"U"** choices made by the computer is exactly the same as the number of **"U"** choices made by the participants in room B. Also, the number of **"D"** choices made by the computer is exactly the same as the number of **"D"** choices made by the room B participants. (Note: while the number of **“U”** numbers and number of **“D”** numbers are the same as in the Room B decisions, which numbers are assigned **“U”** or **“D”** is randomly decided by the computer) For example: if five Room B participants choose **"U"**, then five of the numbers between 1 and 10 are randomly assigned to have the **"U"** payoff, and the remaining five numbers are assigned to the **"D"** payoff. (Note: the numbers used here are only an example and not necessarily representative of Room B decisions)

**Room B (Trustees) Instructions**

Thank you for participating in today’s experiment. You’ve earned a $7 show-up bonus for participating. In reading and following the instructions below, you have the potential to earn significantly more. You have been randomly assigned to **Room B.** You will also be randomly and anonymously assigned to a person in **Room A.** Your counterpart will not be told your name, and you will not be told his/her name.

You will be anonymously assigned to a Room A counterpart who drew your number randomly from a box with the numbers 1 through 10 inside. This person will be your counterpart for the entire experiment. Your counterpart will make a decision that can affect your earnings in today’s experiment. He or she can choose for both of you to be paid $5. Another possibility is that he/she will let you determine both of your payoffs. If he/she chooses this option and you choose **“U”**, then you get paid $15 and he/she gets paid $15. If you choose **“D”**, then you get paid $28 and he/she gets paid $2. Your payoff will be determined in one of these two ways. Your counterpart can choose only one of the earnings methods. We will ask you to make your decision on **“U”** or **“D”** at the same time that your counterpart makes his or her choice. Your decision will only determine your payoff if your counterpart did not choose the option to give you $5.

**Room A (Investors) Quiz Questions: OPTION-TO-KNOW Treatment**

1) You can choose multiple methods to determine your earnings. True / False

2) Your counterpart’s earnings are the same whether you chose to be paid based on your counterpart’s earnings or you chose to be paid based on the computer decision. True / False

3) Your counterpart only gets earnings based on his/her decision if you do not chose for you both to be paid $5. True / False

4) If you choose to be paid based on your counterpart’s decision and he/she chose U, what are your earnings?_______ your counterpart’s?_________

5) If you choose to be paid based on your counterpart’s decision and he/she chose D, what are your earnings?_______ your counterpart’s?_________

6) How many numbers will the computer randomly assign the U value to if exactly 6 Room B counterparts choose U. _________

7) How many numbers will the computer randomly assign the D value to if exactly 6 Room B counterparts choose D. _________

8) The value of the computer decision assigned to a number may not be the same as the value of the decision made by the Room B counterpart with that number. True / False

9) Will you know what your counterpart chose if you choose earnings based on the computer decision? __________

10) Will you know what the computer decision was if you choose earnings based on your counterpart’s decision? __________

**Room B (Trustees) Quiz Questions: All Treatments**

1) If your counterpart chooses for you to both receive $5 and you chose **D** how much are you paid?________ your counterpart? _________

2) If your counterpart chooses for you to both receive $5 and you chose **U** how much are you paid?________ your counterpart? _________

3) If your counterpart chooses to be paid based on your decision and you chose **D** how much are you paid?________ your counterpart?________

4) If your counterpart chooses to be paid based on your decision and you chose **U** how much are you paid?________ your counterpart?________

1. DONTKNOW treatment did not include the second payment choice option, KNOW treatment did not include third payment choice option and did not include the “computer’s decision” paragraph (next page.) [↑](#footnote-ref-2)
