# Supplementary material for: Beneficial Betrayal Aversion
Source: PLoS One. 2011 Mar 14;6(3):e17725. doi: 10.1371/journal.pone.0017725 (PMC3056706; doi:10.1371/journal.pone.0017725)
Supplement: Text S3 — Instructions: DONTKNOW2 (Common Knowledge) Treatment. (DOC) [file pone.0017725.s003.doc]

**Supporting Text S3**

**Instructions (All Subjects): DONTKNOW2 Common Knowledge Treatment[[1]](#footnote-2)**

**Room A Instructions**

Thank you for participating in today’s experiment. You’ve earned a $7 show-up bonus for participating. In reading and following the instructions below, you have the potential to earn significantly more. You have been randomly assigned to **Room A.** You will also be randomly and anonymously assigned to a person in **Room B.** Your counterpart will not be told your name, and you will not be told his/her name.

**How you are matched with your counterpart:**

Each of the 10 Room A persons will be matched with a different Room B counterpart for the entire experiment. The experimenter will bring around a box with the numbers 1 through 10 inside. The number you draw will assign you to one of the 10 counterparts in Room B (B1 through B10 coinciding with the numbers 1 through 10 in the box). The number also matches you with one of the 10 computer number decisions (coinciding with numbers 1 through 10 in the box).

**Your Decision:**

You have two options for how the earnings for you and your counterpart will be determined in today’s experiment. You must choose exactly one of the following two options:

- You receive $5 and your counterpart receives $5.
- Your counterpart is paid according to his/her decision between **“U”** and **“D”**, and you are paid based on a computer’s choice between either **“U”** or **“D”**

You will not be told what the computer’s decision was unless you choose that earnings option.

**Room B Decision:** (The instructions given to your counterpart)

You will be anonymously assigned to a Room A counterpart who drew your number randomly from a box with the numbers 1 through 10 inside. This person will be your counterpart for the entire experiment. Your counterpart will make a decision that can affect your earnings in today’s experiment. He or she can choose for both of you to be paid $5. Another possibility is that he/she will let you determine both of your payoffs. If he/she chooses this option and you choose **“U”**, then you (room B person) get paid $15. If you choose **“D”**, then you (room B person) get paid $28. Your payoff will be determined in one of these two ways. Your counterpart can choose only one of the earnings methods. We will ask you to make your decision on **“U”** or **“D”** at the same time that your counterpart makes his or her choice. Your decision will only determine your payoff if your counterpart did not choose the option to give you $5.

**Computer’s Decision:**
After the Room B participants make their decisions, the computer will assign to each of the ten numbers either **"U"**, meaning a payoff of $15 to you (room A person), or **"D"**, meaning a payoff of $2 to you (room A person). The computer has been programmed to assign dollar values to each of the 10 numbers in the box according to the decisions made by the Room B participants. What this means is that the number of **"U"** choices made by the computer is exactly the same as the number of **"U"** choices made by the participants in room B. Also, the number of **"D"** choices made by the computer is exactly the same as the number of **"D"** choices made by the room B participants. (Note: while the number of **“U”** numbers and number of **“D”** numbers are the same as in the Room B decisions, which numbers are assigned **“U”** or **“D”** is randomly decided by the computer) For example: if five Room B participants choose **"U"**, then five of the numbers between 1 and 10 are randomly assigned to have the **"U"** payoff of $15, and the remaining five numbers are assigned to the **"D"** payoff of $2. (Note: the numbers used here are only an example and not necessarily representative of Room B decisions)

**Room B Instructions**

Thank you for participating in today’s experiment. You’ve earned a $7 show-up bonus for participating. In reading and following the instructions below, you have the potential to earn significantly more. You have been randomly assigned to **Room B.** You will also be randomly and anonymously assigned to a person in **Room A.** Your counterpart will not be told your name, and you will not be told his/her name.

You will be anonymously assigned to a Room A counterpart who drew your number randomly from a box with the numbers 1 through 10 inside. This person will be your counterpart for the entire experiment. Your counterpart will make a decision that can affect your earnings in today’s experiment. He or she can choose for both of you to be paid $5. Another possibility is that he/she will let you determine both of your payoffs. If he/she chooses this option and you choose **“U”**, then you get paid $15. If you choose **“D”**, then you get paid $28. Your payoff will be determined in one of these two ways. Your counterpart can choose only one of the earnings methods. We will ask you to make your decision on **“U”** or **“D”** at the same time that your counterpart makes his or her choice. Your decision will only determine your payoff if your counterpart did not choose the option to give you $5.

1. DONTKNOW2 instructions are identical to the DONTKNOW1 instructions from Appendix A. The only difference in implementation was that both subjects in the role of Investors and subjects in the role of Trustees received both room A and room B instructions, which were read out loud to all subjects in the same room. [↑](#footnote-ref-2)
